# Postpartum Mental Health Care Use Among Parents During Simultaneous Parental Leave

**DOI:** 10.1001/jamanetworkopen.2024.38755

**Published:** 2024-10-14

**Authors:** Helena Honkaniemi, Sol P. Juárez

**Affiliations:** 1 Centre for Health Equity Studies, Stockholm University/Karolinska Institutet, Stockholm, Sweden; 2 Department of Public Health Sciences, Stockholm University, Stockholm, Sweden

## Abstract

**Importance:**

Flexible parental leave schemes can help families balance work, childcare, and postpartum treatment, including treatment for mental health. In Sweden, both parents are eligible to use parental leave simultaneously in the first year after birth; however, the consequences for postpartum mental health care uptake remain underinvestigated.

**Objective:**

To examine the association between parents’ use of simultaneous parental leave and their use of postpartum mental health care in Sweden.

**Design, Setting, and Participants:**

This cohort study obtained baseline data from national registries in Sweden, including live births of singleton offspring between January 1, 2014, to December 31, 2015, and simultaneous parental leave use (any vs none) in the 12 months after birth. The population-based sample was composed of parental dyads (mothers and fathers) who were eligible for simultaneous parental leave and were followed up from January 1, 2015, to December 31, 2016. Statistical analyses were conducted between December 15, 2023, and August 14, 2024.

**Exposure:**

Simultaneous parental leave use up to 12 months post partum.

**Main Outcomes and Measures:**

Total population register data were used to identify maternal and paternal outpatient care visits for mental health (substance use disorder [SUD], mood or affective disorders, and stress-related disorders) and psychotropic prescription dispensations (antidepressants and anxiolytics) during the first postpartum year. Logistic regression was applied to estimate the odds of these outcomes by simultaneous parental leave use, controlling for parents’ age, sociodemographic characteristics, and prebirth mental health care use. Robustness analyses using propensity score matching were performed.

**Results:**

The sample comprised 207 283 parental dyads, of whom 153 342 (74.0%) did not use simultaneous parental leave in the first postpartum year and 53 941 (26.0%) did. Among those who used simultaneous parental leave, the mean (SE) age at childbirth was 29.63 (0.02) years for mothers and 32.80 (0.03) years for fathers. Compared with mothers who did not use parental leave simultaneously with their partners, mothers who did were more likely to receive antidepressant prescriptions in the first postpartum year (odds ratio [OR], 1.07; 95% CI, 1.02-1.11). Fathers who used simultaneous parental leave vs those who did not had increased odds of SUD-related outpatient care visits (OR, 1.10; 95% CI, 1.02-1.20). Longer simultaneous parental leave was associated with greater odds of mental health care use for both parents, and earlier leave was associated with greater odds of health care use by mothers. Findings for mothers, but not fathers, remained significant in robustness analyses using propensity score matching.

**Conclusions and Relevance:**

This cohort study found that simultaneous parental leave use was associated with greater health care uptake for postpartum mental health conditions in both mothers and fathers. This finding supports the need to consider the benefits of generous family policies as well as the risks of structural obstacles to postpartum mental health care access across the world.

## Key Points

**Question:**

What is the association between parental leave used by mothers and fathers simultaneously and their postpartum mental health care use?

**Findings:**

In this cohort study of 207 283 parent dyads in Sweden, simultaneous parental leave use was associated with increased odds of mental health care uptake, specifically antidepressant prescriptions for mothers and substance use disorder–related outpatient services for fathers. Earlier simultaneous parental leave use was only associated with increased maternal health care uptake, whereas prolonged leave was associated with greater uptake for both parents.

**Meaning:**

The findings suggest that flexible family policies provide parents the opportunity to seek necessary mental health treatment during the critical postpartum period.

## Teaser

This cohort study assesses the association between concurrent postpartum work leave among parents and their likelihood to participate in mental health services in Sweden.

## Introduction

The transition to parenthood is a highly sensitive period that often manifests in elevated affective and stress-related symptoms in both parents. Worldwide, evidence suggests that in the year after birth, up to 17% of mothers and 9% of fathers experience postpartum depression (PPD),^1,2^ with similar levels of postpartum anxiety,^3,4^ and other comorbid mental health symptoms (including substance use–related behaviors).^5,6,7^ These outcomes may be attributed to hormonal and physical changes; the stress of adopting new parenting roles, routines, and dynamics; and underlying mental health conditions.^1,2,8^ Yet, given the already high demands of balancing work and childcare, in addition to persisting structural obstacles to health care, parents may lack the opportunity to seek necessary mental health treatment.^8,9^

Generous parental leave has been found to be protective for the postpartum mental health of both mothers and fathers.^10^ Most efforts to incentivize leave participation, such as through nontransferable quotas, have been associated with improved mental health and related behavioral outcomes in affected parents.^7,11^ Yet, little is known about the consequences of a flexible parental leave that grants parents the freedom to choose their level of involvement, especially to address unmet mental health needs.

Paid simultaneous parental leave, which allows parents to be remunerated for being on leave at the same time, is one form of such flexibility. In Sweden, parents have long been entitled to extensive, job-protected, and well-remunerated leave (currently, up to 480 days per child) that can be shared as they see fit (except for 90 days, which are reserved for each parent). While nonbirthing partners have been eligible to be reimbursed with a temporary allowance for up to 10 days at home in the 2 months after birth, it was not until 2012 that all parents gained the option to use paid leave simultaneously for up to 30 days in the child’s first year of life.^12^ Beyond potentially alleviating postpartum stress levels through improved work-life balance^13,14^ and greater infant bonding,^15,16^ simultaneous time at home could allow both parents to seek care for existing mental health concerns. While this opportunity is supported by evidence on simultaneous parental leave eligibility and mental health care–seeking in mothers,^17^ no study has investigated the actual role that simultaneous parental leave plays in both maternal and paternal mental health treatment. Thus, this study aimed to examine the association between parents’ use of simultaneous parental leave and their use of postpartum mental health care in Sweden.

## Methods

This cohort study was conducted as part of the Unintended Health Consequences of Swedish Parental Leave Policy (ParLeHealth) research project based on a peer-reviewed project protocol.^18^ The Swedish Ethical Review Authority approved the study and waived the informed consent requirement due to the pseudonymized nature of the data. We followed the Strengthening the Reporting of Observational Studies in Epidemiology (STROBE) reporting guideline.

### Study Data

Data were linked across the Swedish total population registers through pseudonymized personal identification numbers. Using the Medical Birth Register,^19^ we first identified all live births in Sweden from January 1, 2014, to December 31, 2015 (n = 226 016), removing multiple births, subsequent births to the same parents within the study period, and births leading to adoption (eFigure in Supplement 1). To ensure that both parents were entitled to use parental leave, we then excluded observations with missing information on the father, with missing information on fathers who were not residing in Sweden at the time of birth, and wherein either parent was not residing in Sweden for the full follow-up period (January 1, 2015, to December 31, 2016). Additionally, we excluded observations if the father had died before birth or if either parent or child died within the first postpartum year.

### Parental Leave Exposures and Mental Health Outcomes

We identified simultaneous parental leave use (any vs none) in the 12 months post partum using the Swedish Social Insurance Agency Microdata Register. We specified the length (1-15 days or 16-30 days) and timing (0-5 months or 6-12 months post partum) of simultaneous parental leave use within this period.

In Sweden, new parents are invited to routine checkups with Child Health Services (CHS), including screenings for PPD and related symptoms for mothers around 2 months post partum^20,21,22,23^ and less commonly screenings for nonbirthing partners between 3 and 5 months post partum.^21,23,24,25^ Up to 12% of mothers and 6% of fathers in Sweden have been shown to exhibit PPD-related symptoms.^24^ If symptoms are detected, parents may receive supportive counseling within CHS or be referred elsewhere. Psychotropic prescriptions are available through primary care, whereas both psychotherapy and psychotropic prescriptions are available through specialist outpatient care for more severe symptoms.^20^

First, using the National Patient Register,^26^ we identified whether parents had any outpatient visits for mental and behavioral disorders associated with psychoactive substance use disorder (SUD) with *International Statistical Classification of Diseases and Related Health Problems, Tenth Revision* (*ICD-10*) codes F10 to F19; mood or affective disorders with *ICD-10* codes F30 to F39; or neurotic, stress-related, and somatoform disorders with *ICD-10* codes F40 to F49 in the first postpartum year. Second, using the Prescribed Drug Register,^27^ we captured parental prescriptions for antidepressants (with Anatomical Therapeutic Chemical [ATC] code N06A) and anxiolytics (with ATC code N05B) in the year after childbirth, specifically dispensed prescriptions made via all treatment channels (primary, specialist outpatient, and inpatient care). Third, to assess potential selection into simultaneous parental leave use, we included a dichotomous covariate of parents’ prebirth mental health care use (outpatient visits for mental health or psychotropic prescriptions) up to 2 years before childbirth.

### Covariates

Using data from the Longitudinal Integrated Database for Health Insurance and Labour Market Studies,^28^ we specified parental age (calendar year of birth), educational level (low: up to 2 years of upper secondary education; medium: up to 2 years of university college; high: graduate or postgraduate studies; or missing data; calendar year of birth), annual labor income (quintiles, in Swedish kronor; calendar year before birth), civil status (married or cohabiting vs single or noncohabiting; calendar year after birth), and nativity (Swedish born or non–Swedish born). These factors have been associated with different levels of simultaneous parental leave use in Sweden.^29^

### Statistical Analysis

Logistic regression analyses were used to estimate the odds of mental health care uptake by simultaneous parental leave use in the first postpartum year. Models were stratified by parent, with stepwise adjustment for both parents’ age, socioeconomic and demographic factors, and individual prebirth mental health care use. Subgroup analyses were conducted to further explore parental strategies regarding the length and timing of simultaneous parental leave use.

As a robustness check, we applied propensity score matching to the main models to further account for potential confounding through selection into simultaneous parental leave use.^30^ The propensity score or estimated probability of treatment (simultaneous parental leave use) was calculated based on preselected covariates (parental age, educational level, annual labor income, nativity, and prebirth mental health care use) first before matching individuals in the treatment and control groups 1:1 based on their propensity scores using the nearest-neighbor method. After matching, the mean difference in each outcome between groups was estimated (using Stata command teffects psmatch) and checked for covariate balance (using teffects summarize).

As a sensitivity analysis, we excluded parents with prebirth mental health care use to explore incident health concerns. Parents coded as single or noncohabiting in the calendar year after birth were also excluded to assess whether parents were entitled and inclined to use simultaneous parental leave. Statistical analyses were conducted between December 15, 2023, and August 14, 2024, using Stata, version 17.0 (StataCorp LLC).

## Results

In the sample of 207 283 parental dyads (mothers and fathers), 153 342 (74.0%) did not use simultaneous parental leave in the first postpartum year. Of the 53 941 parents (26.0%) who used simultaneous parental leave, the mean (SE) age at childbirth was 29.63 (0.02) years for mothers and 32.80 (0.03) years for fathers, 44.2% of mothers and 31.4% of fathers had a high educational level, and 23.6% of mothers and 21.7% of fathers were in the top quintile of labor income (Table 1 provides additional parental data). Among these parents, 13.0% of mothers and 8.0% of fathers used outpatient mental health care services, while 14.2% and 9.2%, respectively, received psychotropic prescriptions. Simultaneous parental leave was used for up to 15 of the available 30 days among 62.2% of parents, with 40.9% of parents using these days in both the first and second half of the postpartum year.

**Table 1.** Descriptive Characteristics of Parents of Singleton Offspring Born From January 1, 2014, to December 31, 2015, in Sweden

Characteristic	Simultaneous parental leave use, No. (%)			
	No (n = 153 342)		Yes (n = 53 941)	
	Mothers	Fathers	Mothers	Fathers
Mental health care outcomes				
Outpatient care use with mental health diagnosis (from birth to 12 mo post partum)	17 806 (11.6)	11 967 (7.8)	7033 (13.0)	4327 (8.0)
Disorders due to psychoactive SUD	1266 (0.8)	2200 (1.4)	546 (1.0)	867 (1.6)
Mood or affective disorders	4964 (3.2)	2496 (1.6)	2019 (3.7)	909 (1.7)
Neurotic, stress-related, and somatoform disorders	8063 (5.3)	4720 (3.1)	3152 (5.8)	1638 (3.0)
Psychotropic prescriptions (from birth to 12 mo post partum)	19 054 (12.4)	14 180 (9.3)	7664 (14.2)	4982 (9.2)
Antidepressants	13 990 (9.1)	9620 (6.3)	5815 (10.8)	3438 (6.4)
Anxiolytics	7290 (4.8)	6361 (4.2)	2781 (5.2)	2158 (4.0)
Prebirth mental health care use (up to 24 mo before birth)	17 714 (11.6)	12 921 (8.4)	7181 (13.3)	4511 (8.4)
Simultaneous parental leave characteristics				
Length of leave, d				
1-15	NA	NA	NA	33 532 (62.2)
16-30	NA	NA	NA	20 409 (37.8)
Timing of leave post partum, mo				
Only 0-5	NA	NA	NA	11 501 (21.3)
Only 6-12	NA	NA	NA	20 376 (37.8)
Both 0-5 and 6-12	NA	NA	NA	22 064 (40.9)
Sociodemographic characteristics				
Age, mean (SE), y	30.59 (0.01)	34.16 (0.02)	29.63 (0.02)	32.80 (0.03)
Educational level^a^				
Low	16 864 (11.0)	20 327 (13.3)	4163 (7.7)	5524 (10.2)
Medium	59 266 (38.7)	78 986 (48.3)	25 401 (47.1)	31 011 (57.5)
High	73 981 (48.3)	56 644 (36.9)	23 985 (44.5)	16 961 (31.4)
Missing data	3231 (2.1)	2385 (1.6)	542 (1.0)	445 (0.8)
Annual labor income, mean (SE), Sk	2017.35 (4.32)	3049.93 (6.05)	2102.84 (6.39)	3200.13 (7.44)
Bottom quintile	40 899 (26.7)	42 323 (27.6)	10 256 (19.0)	8958 (16.6)
Top quintile	38 399 (25.0)	39 551 (25.8)	12 732 (23.6)	11 724 (21.7)
Civil status: separated	NA	18 476 (12.1)	NA	4332 (8.0)
Nativity: non–Swedish born	43 523 (28.8)	45 061 (29.4)	9745 (18.1)	9696 (18.0)

Abbreviations: NA, not applicable; Sk, Swedish krona (to convert to US dollars, multiply by 0.098); SUD, substance use disorder.

^a^ Low: up to 2 years of upper secondary education; medium: up to 2 years of university college; and high: graduate or postgraduate studies.

Logistic regression analyses suggested that simultaneous parental leave use was associated with increased odds of outpatient mental health care visits and psychotropic prescriptions among mothers in the first postpartum year (Figure, A; Table 2 [model 1]). In particular, mothers who used simultaneous parental leave had greater odds of outpatient visits for mood or affective disorders (odds ratio [OR], 1.15; 95% CI, 1.09-1.22) and antidepressant prescriptions (OR, 1.20; 95% CI, 1.17-1.24) than those who did not. Results were slightly attenuated after controlling for parental characteristics (model 2) and fully attenuated when further controlling for previous mental health care use (model 3) except antidepressant prescriptions (OR, 1.07; 95% CI, 1.02-1.11). Analyses for fathers suggested no differences in postpartum mental health care with simultaneous parental leave use (Figure, B; Table 2 [model 1]) but slightly elevated risks of SUD-related and mood or affective disorder visits as well as antidepressant prescriptions after controlling for parental characteristics (model 2). Further controlling for prebirth mental health care use attenuated all results except for SUD-related visits (OR, 1.10; 95% CI, 1.02-1.20; model 3).

**Figure.**
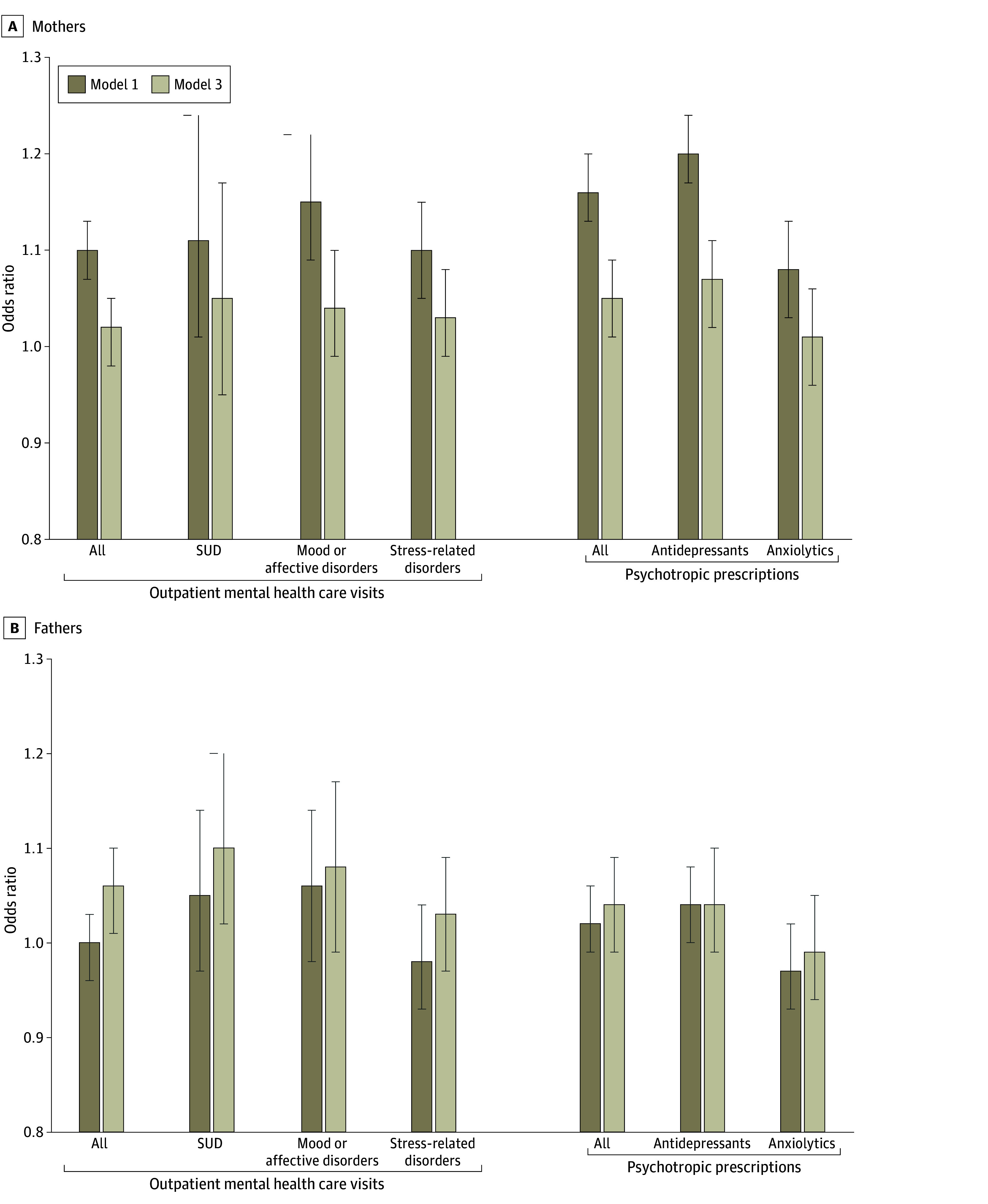
Mental Health Care Uptake by Simultaneous Parental Leave Use Estimates were adjusted as follows: model 1, by maternal and paternal age at childbirth; model 3, by model 1 plus maternal and paternal educational level, annual income, nativity, and maternal or paternal mental health care use before childbirth. For all estimates, see Table 2. Error bars represent 95% CIs. SUD indicates substance use disorder.

**Table 2.** Main Analyses: Simultaneous Parental Leave Use and Parental Use of Postpartum Mental Health Care^a^

	Parent dyads, OR (95% CI) (N = 207 283)					
	Mothers			Fathers		
	Model 1	Model 2	Model 3	Model 1	Model 2	Model 3
By any simultaneous parental leave use (reference: no leave use)						
Outpatient mental health care visits	1.10 (1.07-1.13)	1.07 (1.04-1.10)	1.02 (0.98-1.05)	1.00 (0.96-1.03)	1.06 (1.02-1.10)	1.06 (1.01-1.10)
SUD	1.11 (1.01-1.24)	1.07 (0.96-1.18)	1.05 (0.95-1.17)	1.05 (0.97-1.14)	1.09 (1.01-1.19)	1.10 (1.02-1.20)
Mood or affective disorders	1.15 (1.09-1.22)	1.10 (1.05-1.16)	1.04 (0.99-1.10)	1.06 (0.98-1.14)	1.09 (1.01-1.18)	1.08 (0.99-1.17)
Stress-related disorders	1.10 (1.05-1.15)	1.09 (1.04-1.14)	1.03 (0.99-1.08)	0.98 (0.93-1.04)	1.04 (0.98-1.11)	1.03 (0.97-1.09)
Psychotropic prescriptions	1.16 (1.13-1.20)	1.12 (1.08-1.15)	1.05 (1.01-1.09)	1.02 (0.99-1.06)	1.06 (1.02-1.10)	1.04 (0.99-1.09)
Antidepressants	1.20 (1.17-1.24)	1.13 (1.10-1.17)	1.07 (1.02-1.11)	1.04 (1.00-1.08)	1.06 (1.02-1.11)	1.04 (0.99-1.10)
Anxiolytics	1.08 (1.03-1.13)	1.07 (1.03-1.13)	1.01 (0.96-1.06)	0.97 (0.93-1.02)	1.02 (0.98-1.08)	0.99 (0.94-1.05)

Abbreviations: OR, odds ratio; SUD, substance use disorder.

^a^ Estimates were adjusted as follows: model 1, by maternal and paternal age at childbirth; model 2, by model 1 plus maternal and paternal educational level, annual income, and nativity; and model 3, by model 2 plus maternal or paternal mental health care use before childbirth.

Subgroup analyses by length of simultaneous parental leave use indicated variation in mental health care outcomes (Table 3). For parents using up to 15 days of simultaneous parental leave compared with those not using simultaneous parental leave, mothers had greater odds of antidepressant prescription receipt (OR, 1.07; 95% CI, 1.02-1.12), whereas those using more than 15 days of simultaneous parental leave had greater odds of maternal outpatient visits for SUD (OR, 1.16; 95% CI, 1.01-1.33) and stress-related disorders (OR, 1.09; 95% CI, 1.02-1.16; model 3). Meanwhile, up to 15 days of simultaneous parental leave use was associated with decreased mental health care use among fathers (models 1 and 2), which was attenuated after further adjustment (model 3) (Table 3). Sixteen to 30 days of leave were associated with consistently elevated odds of paternal outpatient mental health care visits (OR, 1.14; 95% CI, 1.08-1.21) and psychotropic prescriptions (OR, 1.08; 95% CI, 1.01-1.15; model 3).

**Table 3.** Subgroup Analyses: Length of Simultaneous Parental Leave and Parental Use of Postpartum Mental Health Care^a^

Length of simultaneous parental leave (reference: 0 d)	Parent dyads, OR (95% CI) (N = 207 283)					
	Mothers			Fathers		
	Model 1	Model 2	Model 3	Model 1	Model 2	Model 3
**1-15 d**						
Outpatient mental health care visits	1.02 (0.98-1.06)	1.02 (0.99-1.06)	0.98 (0.94-1.02)	0.87 (0.83-0.91)	0.98 (0.93-1.03)	1.00 (0.94-1.05)
SUD	0.98 (0.87-1.12)	0.98 (0.86-1.12)	0.97 (0.85-1.11)	0.86 (0.78-0.96)	0.97 (0.87-1.08)	1.00 (0.89-1.11)
Mood or affective disorders	1.09 (1.02-1.17)	1.07 (0.99-1.14)	1.02 (0.95-1.09)	0.92 (0.83-1.01)	0.99 (0.89-1.09)	1.00 (0.90-1.11)
Stress-related disorders	1.02 (0.97-1.08)	1.04 (0.99-1.10)	1.00 (0.94-1.06)	0.90 (0.84-0.97)	1.00 (0.93-1.08)	1.01 (0.94-1.09)
Psychotropic prescriptions	1.13 (1.09-1.17)	1.10 (1.06-1.14)	1.04 (0.99-1.10)	0.93 (0.89-0.97)	1.00 (0.96-1.05)	1.01 (0.95-1.07)
Antidepressants	1.18 (1.14-1.23)	1.12 (1.08-1.16)	1.07 (1.02-1.12)	0.96 (0.91-1.01)	1.01 (0.96-1.07)	1.03 (0.96-1.09)
Anxiolytics	1.03 (0.98-1.09)	1.05 (0.99-1.11)	1.00 (0.94-1.06)	0.86 (0.82-0.92)	0.94 (0.88-1.01)	0.94 (0.87-1.01)
**16-30 d**						
Outpatient mental health care visits	1.23 (1.18-1.28)	1.14 (1.09-1.19)	1.08 (1.03-1.14)	1.21 (1.15-1.27)	1.17 (1.11-1.23)	1.14 (1.08-1.21)
SUD	1.32 (1.16-1.52)	1.18 (1.03-1.35)	1.16 (1.01-1.33)	1.35 (1.22-1.50)	1.25 (1.12-1.39)	1.23 (1.11-1.38)
Mood or affective disorders	1.25 (1.16-1.35)	1.16 (1.08-1.25)	1.08 (0.99-1.17)	1.29 (1.16-1.43)	1.24 (1.11-1.38)	1.18 (1.06-1.32)
Stress-related disorders	1.23 (1.16-1.30)	1.16 (1.09-1.23)	1.09 (1.02-1.16)	1.12 (1.03-1.21)	1.10 (1.01-1.19)	1.04 (0.96-1.14)
Psychotropic prescriptions	1.22 (1.17-1.27)	1.15 (1.10-1.20)	1.05 (0.99-1.11)	1.17 (1.11-1.22)	1.15 (1.09-1.21)	1.08 (1.01-1.15)
Antidepressants	1.24 (1.18-1.30)	1.16 (1.10-1.21)	1.06 (0.99-1.12)	1.17 (1.11-1.24)	1.14 (1.08-1.21)	1.06 (0.98-1.14)
Anxiolytics	1.16 (1.08-1.23)	1.11 (1.04-1.19)	1.02 (0.95-1.10)	1.15 (1.08-1.24)	1.15 (1.07-1.23)	1.06 (0.99-1.15)

Abbreviations: OR, odds ratio; SUD, substance use disorder.

^a^ Estimates were adjusted as follows: model 1, by maternal and paternal age at childbirth; model 2, by model 1 plus maternal and paternal educational level, annual income, and nativity; and model 3, by model 2 plus maternal or paternal mental health care use before childbirth.

Subgroup analyses of the timing of simultaneous parental leave and mental health care use in 6-month intervals were also conducted. Overall, simultaneous parental leave in the first 6 months post partum was associated with elevated odds of maternal mood or affective disorder visits and psychotropic prescriptions during that same period, although only prescription odds remained high after adjustment (OR, 1.10; 95% CI, 1.03-1.17) (Table 4 [model 3]). Simultaneous parental leave use in the second half of the postpartum year was also associated with greater odds of antidepressant prescription receipt, although this was attenuated when controlling for prebirth mental health care use. Among fathers, simultaneous parental leave use in both the first and second half of the postpartum year was associated with consistently decreased odds of outpatient visits (eg, 0-5 months, mood or affective disorders: OR, 0.62; 95% CI, 0.48-0.79) (Table 4 [model 3]).

**Table 4.** Subgroup Analyses: Timing of Simultaneous Parental Leave and Parental Use of Postpartum Mental Health Care^a^

Timing of simultaneous parental leave (reference: no leave use)	Parent dyads, OR (95% CI) (N = 207 283)					
	Mothers			Fathers		
	Model 1	Model 2	Model 3	Model 1	Model 2	Model 3
**0-5 mo post partum**						
Outpatient mental health care visits	1.14 (1.05-1.23)	1.09 (1.01-1.19)	1.01 (0.93-1.10)	0.76 (0.69-0.85)	0.81 (0.73-0.91)	0.78 (0.69-0.87)
SUD	0.76 (0.46-1.27)	0.78 (0.47-1.31)	0.75 (0.45-1.26)	0.84 (0.66-1.08)	0.95 (0.75-1.23)	0.96 (0.75-1.23)
Mood or affective disorders	1.18 (1.04-1.34)	1.12 (0.98-1.27)	1.02 (0.89-1.16)	0.63 (0.49-0.80)	0.64 (0.50-0.82)	0.62 (0.48-0.79)
Stress-related disorders	1.13 (0.99-1.28)	1.10 (0.97-1.25)	1.02 (0.89-1.16)	0.69 (0.57-0.85)	0.77 (0.63-0.94)	0.74 (0.61-0.91)
Psychotropic prescriptions	1.29 (1.22-1.36)	1.18 (1.12-1.25)	1.10 (1.03-1.17)	1.01 (0.95-1.08)	1.01 (0.95-1.08)	0.95 (0.88-1.02)
Antidepressants	1.30 (1.23-1.38)	1.18 (1.11-1.25)	1.09 (1.02-1.16)	1.02 (0.95-1.10)	1.01 (0.94-1.09)	0.95 (0.87-1.03)
Anxiolytics	1.28 (1.16-1.42)	1.22 (1.11-1.35)	1.16 (1.05-1.28)	0.93 (0.83-1.03)	0.95 (0.86-1.06)	0.92 (0.82-1.02)
**6-12 mo post partum**						
Outpatient mental health care visits	0.93 (0.85-1.01)	0.99 (0.90-1.07)	0.95 (0.87-1.04)	0.68 (0.61-0.76)	0.80 (0.71-0.89)	0.80 (0.72-0.90)
SUD	0.71 (0.46-1.10)	0.89 (0.57-1.38)	0.87 (0.55-1.36)	0.52 (0.40-0.69)	0.64 (0.48-0.84)	0.66 (0.49-0.87)
Mood or affective disorders	1.01 (0.88-1.16)	1.02 (0.89-1.17)	0.98 (0.85-1.13)	0.70 (0.57-0.87)	0.77 (0.62-0.95)	0.77 (0.62-0.96)
Stress-related disorders	0.92 (0.80-1.06)	0.96 (0.84-1.11)	0.94 (0.81-1.08)	0.72 (0.60-0.87)	0.86 (0.71-1.04)	0.87 (0.72-1.06)
Psychotropic prescriptions	1.11 (1.06-1.17)	1.07 (1.02-1.13)	1.04 (0.99-1.10)	0.92 (0.87-0.99)	0.96 (0.90-1.02)	0.95 (0.88-1.01)
Antidepressants	1.13 (1.07-1.19)	1.07 (1.02-1.13)	1.04 (0.98-1.10)	0.94 (0.88-1.01)	0.97 (0.90-1.04)	0.95 (0.88-1.03)
Anxiolytics	1.01 (0.92-1.11)	1.02 (0.93-1.12)	1.00 (0.91-1.10)	0.86 (0.77-0.95)	0.91 (0.82-1.01)	0.91 (0.82-1.01)

Abbreviations: OR, odds ratio; SUD, substance use disorder.

^a^ Estimates were adjusted as follows: model 1, by maternal and paternal age at childbirth; model 2, by model 1 plus maternal and paternal educational level, annual income, and nativity; and model 3, by model 2 plus maternal or paternal mental health care use before childbirth

Robustness analyses based on propensity score matching showed elevated probabilities of antidepressant prescriptions among mothers (ATET [average treatment effect on the treated] coefficient, 0.00468; 95% CI, 0.00190-0.00744) but no differences in any of the outcomes among fathers, stratified by simultaneous parental leave use (eTable 1 in Supplement 1). Postestimation checks suggested greater balance in all covariates after matching (eTable 2 in Supplement 1). Sensitivity analyses excluding individuals with prebirth mental health care use revealed greater odds of outpatient mental health care visits for stress-related disorders (OR, 1.11; 95% CI, 1.04-1.18) and receipt of either psychotropic prescription among mothers (antidepressants: OR, 1.10 [95% CI, 1.04-1.17]; anxiolytics: OR, 1.10 [95% CI, 1.02-1.18]), stratified by simultaneous parental leave use (eTable 3A in Supplement 1). Fathers using simultaneous parental leave had elevated odds of outpatient visits for SUD (OR, 1.19; 95% CI, 1.07-1.32) and mood or affective disorders (OR, 1.14; 95% CI, 1.02-1.28) (eTable 3B in Supplement 1). Excluding individuals who were coded as single or noncohabiting in the calendar year after birth revealed increased odds of SUD-related visits (OR, 1.13; 95% CI, 1.00-1.27) and antidepressant prescriptions among mothers (OR, 1.05; 95% CI, 1.01-1.10) as well as SUD-related visits (OR, 1.24; 95% CI, 1.12-1.37) and mood or affective disorder visits among fathers (OR 1.11; 95% CI, 1.01-1.21) (eTable 4 in Supplement 1).

## Discussion

The findings showed elevated odds of antidepressant prescription receipt among mothers and SUD-related outpatient care visits among fathers who used parental leave simultaneously. Analyses by length and timing of simultaneous parental leave suggested that parents who used more than 15 days of simultaneous parental leave were more likely to seek outpatient mental health care services than those who did not use such leave. Simultaneous parental leave use in the first half of the postpartum year had a greater-magnitude association with mental health care outcomes among mothers, whereas fathers had decreased mental health care uptake during this period. Overall, the findings appeared to be partly confounded by prebirth mental health care use. The results suggest that simultaneous parental leave could be an effective strategy for addressing preexisting and incident mental health conditions.

A recent systematic review concluded that generous parental leave plays a protective role in parental mental health, but it did not find evidence of whether these benefits were associated with simultaneous or sequential parental leave use.^10^ The current study indicated that even only 1 month of leave flexibility can be beneficial for parental mental health by allowing partners to seek care during the postpartum period. Although we were unable to identify whether higher use of mental health care services reflects greater need of or access to these services, the voluntary nature of simultaneous parental leave and the relatively short follow-up suggest that the findings better reflect increased access to these services rather than adverse changes in underlying mental health. These results highlight the importance of considering not only the structural determinants of postpartum mental health problems among parents but also the unintended health consequences of the introduction and expansion of parental leave systems worldwide.

The findings of the current study are partly supported by a previous investigation of the health outcomes of the 2012 parental leave reform in Sweden, which introduced the 30 days of simultaneous parental leave we investigated.^17^ The previous quasi-experimental study, which did not capture actual (but rather eligibility for) simultaneous parental leave use nor the full period of eligibility up to 1 year post partum, revealed decreased maternal risks of anxiolytic prescription receipt in the first 3 months after birth. While this finding aligns with that in the subgroup analyses we conducted of up to 6 months post partum, we also observed elevated odds of outpatient mental health care use among mothers by more simultaneous parental leave claimed and by later uptake. These differences suggest that mothers may resort to seeking outpatient care services only for more serious or prolonged postpartum mood or affective disorder symptoms.

Meanwhile, previous evidence on the implications of simultaneous parental leave for paternal mental health care use is scarce. In Sweden, most research has focused on investigating quota-based efforts to promote fathers’ participation in parental leave,^31^ with evidence of lower risks of psychiatric and alcohol use–related hospitalizations among fathers following these quotas.^7,11^ Findings of the present study suggest that increasing leave flexibility could be a factor in higher paternal outpatient mental health care use, possibly reducing the chances of experiencing severe psychiatric outcomes in the first place. Furthermore, the study supports parents’ strategic approaches to flexible leave use, with those using up to 15 days of leave appearing to prioritize maternal health needs and those using more than 15 days emphasizing both parents’ needs. Increased paternal outpatient visits for SUD, a common externalization of underlying mood or affective disorder symptoms among men,^6^ suggest that simultaneous parental leave can be used for fathers’ unique mental health concerns.

The observed association between simultaneous parental leave and the investigated outcomes appeared to be partially confounded by prebirth mental health care use, a factor in postpartum mental health.^1,2,32^ However, in response to common concerns regarding health selection,^10^ controlling for and excluding individuals with earlier mental health care use did not attenuate all findings. Further excluding parents who were no longer in a registered partnership in the year after birth revealed increased odds of antidepressant prescriptions (for mothers) and SUD-related outpatient care (for fathers) compared with the main analyses. This finding suggests possible confounding by noneligible parents: those who were no longer cohabiting during the first postpartum year and thus not entitled to use simultaneous parental leave. Later dissolution of the partnership may have also been indicative of low relationship quality underlying both a lower inclination to share leave^33,34^ and poor mental health.^1,2,32^

To date, Sweden is one of a few countries that allow parents to be on paid leave at the same time,^35^ and in July 2024, Sweden even extended the simultaneous parental leave period to 60 days.^36^ Nevertheless, this study has implications across all contexts, highlighting the importance of parents having the opportunity to effectively share work, parenting, and health care responsibilities in the critical first postpartum year. Findings of this study emphasize that mothers in particular need time to rest, recover, and seek necessary care in at least the first 6 months post partum, which may be difficult when they are primarily concerned with childcare, not to mention early return to work.^8,9^ The findings also suggest that fathers’ mental health needs should be prioritized when designing family policies and when considering postpartum health care access.

### Limitations

Despite its use of robust longitudinal total population data, this study has some limitations. First, due to its observational nature, the association between simultaneous parental leave and mental health care use may be altered by unmeasured confounding, with some parents being more inclined to use simultaneous parental leave and to have mental health concerns in the first place. Due to a lack of data on simultaneous parental leave uptake around the 2012 reform, more advanced quasi-experimental methods (eg, instrumental variable analyses) were not feasible.^37^ Instead, we aimed to address any selection effects through stepwise controls and propensity score matching.^30^ Furthermore, the study focus remained on the association of simultaneous parental leave use with treatment rather than with health in and of itself. Due to the lack of CHS-based and primary care–based data, we may have underestimated postpartum mental health concerns in this population if parents sought primary care independently of CHS-based referrals or were not prescribed medication. For ease of interpretation, we investigated variables aggregated to the first postpartum year, or stratified into 6-month intervals, without specifying whether simultaneous parental leave and mental health care were actually co-occurring. We could not ascertain whether simultaneous parental leave use had a long-term role in maternal and paternal mental health due to a lack of up-to-date data. Additionally, although the findings were limited to heterosexual partners, in future research, it is important to study parents of different sexual orientations and gender identities.

## Conclusions

This cohort study found increased odds of mental health care uptake, specifically antidepressant prescriptions for mothers and SUD-related outpatient services for fathers, when parents took simultaneous leave. This finding suggests that flexible parental leave schemes are crucial for promoting postpartum mental health care among both mothers and fathers. Parents appeared to have initially used simultaneous parental leave days to address postpartum symptoms among mothers but, with longer uptake, were additionally shown to use leave to promote fathers’ health care use. For the postpartum health of parents, policymakers across the world should consider the benefits of generous family policies as well as the risks of existing structural obstacles to mental health care access.^8,9^

## Notes

Supplement 1. **Figure.** Flow Diagram of Study Population Selection Process **eTable 1.** Robustness Analysis: Propensity Score Matching Results of Association Between Simultaneous Parental Leave and Parents’ Mental Healthcare Use **eTable 2.** Robustness Analysis: Propensity Score Matching Postestimation Covariate Balance Summary **eTable 3.** Sensitivity Analysis: Simultaneous Parental Leave and Parents’ Mental Healthcare Use, Excluding Individuals With Mental Healthcare Use Before Birth **eTable 4.** Sensitivity Analysis: Simultaneous Parental Leave and Parents’ Mental Healthcare Use, Excluding Single and Non-Cohabiting Individuals in the Year After Birth

Supplement 2.
Data Sharing Statement

## References

[1] Liu X, Wang S, Wang G. Prevalence and risk factors of postpartum depression in women: a systematic review and meta-analysis. J Clin Nurs. 2022;31(19-20):2665-2677. doi:10.1111/jocn.16121 34750904

[2] Rao WW, Zhu XM, Zong QQ, . Prevalence of prenatal and postpartum depression in fathers: a comprehensive meta-analysis of observational surveys. J Affect Disord. 2020;263:491-499. doi:10.1016/j.jad.2019.10.030 31757623

[3] Leach LS, Poyser C, Cooklin AR, Giallo R. Prevalence and course of anxiety disorders (and symptom levels) in men across the perinatal period: a systematic review. J Affect Disord. 2016;190:675-686. doi:10.1016/j.jad.2015.09.063 26590515

[4] Goodman JH, Watson GR, Stubbs B. Anxiety disorders in postpartum women: a systematic review and meta-analysis. J Affect Disord. 2016;203:292-331. doi:10.1016/j.jad.2016.05.033 27317922

[5] Chapman SLC, Wu LT. Postpartum substance use and depressive symptoms: a review. Women Health. 2013;53(5):479-503. doi:10.1080/03630242.2013.804025 23879459 PMC3742364

[6] Dimova ED, McGarry J, McAloney-Kocaman K, Emslie C. Exploring men’s alcohol consumption in the context of becoming a father: a scoping review. Drugs Educ Prev Policy. 2022;29(6):643-654. doi:10.1080/09687637.2021.1951669

[7] Honkaniemi H, Juárez SP. Alcohol-related morbidity and mortality by fathers’ parental leave: a quasi-experimental study in Sweden. Addiction. 2024;119(2):301-310. doi:10.1111/add.16354 37798819

[8] Wisner KL, Murphy C, Thomas MM. Prioritizing maternal mental health in addressing morbidity and mortality. JAMA Psychiatry. 2024;81(5):521-526. doi:10.1001/jamapsychiatry.2023.5648 38381408

[9] Shuffrey LC, Thomason ME, Brito NH. Improving perinatal maternal mental health starts with addressing structural inequities. JAMA Psychiatry. 2022;79(5):387-388. doi:10.1001/jamapsychiatry.2022.0097 35262622 PMC9081213

[10] Heshmati A, Honkaniemi H, Juárez SP. The effect of parental leave on parents’ mental health: a systematic review. Lancet Public Health. 2023;8(1):e57-e75. doi:10.1016/S2468-2667(22)00311-5 36603912

[11] Honkaniemi H, Katikireddi SV, Rostila M, Juárez SP. Psychiatric consequences of a father’s leave policy by nativity: a quasi-experimental study in Sweden. J Epidemiol Community Health. 2022;76(4):367-373. doi:10.1136/jech-2021-217980 34635548 PMC8921563

[12] Duvander AZ, Löfgren N. Sweden country note. In: Blum S, Dobrotić I, Kaufman G, Koslowski A, Moss P, eds. *International Review of Leave Policies and Research*. International Network on Leave Policies & Research; 2023. Accessed April 5, 2024. https://www.leavenetwork.org/fileadmin/user_upload/k_leavenetwork/annual_reviews/2023/Sweden2023.pdf

[13] Lidbeck M, Bernhardsson S, Tjus T. Division of parental leave and perceived parenting stress among mothers and fathers. J Reprod Infant Psychol. 2018;36(4):406-420. doi:10.1080/02646838.2018.1468557 29764194

[14] Karimi A, Lindahl E, Skogman Thoursie P. Labour supply responses to paid parental leave. IFAU Working Paper. 2012. Accessed April 5, 2024. https://www.ifau.se/Forskning/Publikationer/Working-papers/2012/Labour-supply-responses-to-paid-parental-leave/

[15] Kerstis B, Aarts C, Tillman C, . Association between parental depressive symptoms and impaired bonding with the infant. Arch Womens Ment Health. 2016;19(1):87-94. doi:10.1007/s00737-015-0522-3 25854998

[16] Schaber R, Kopp M, Zähringer A, Mack JT, Kress V, Garthus-Niegel S. Paternal leave and father-infant bonding: findings from the population-based cohort study DREAM. Front Psychol. 2021;12:668028. doi:10.3389/fpsyg.2021.668028 34149562 PMC8212974

[17] Persson P, Rossin-Slater M. When dad can stay home: fathers’ workplace flexibility and maternal health. National Bureau of Economic Research working paper 25902. Revised October 2019. Accessed April 5, 2024. https://www.nber.org/papers/w25902.pdf 10.1257/app.20220400 PMC12463307 41019476

[18] Juárez SP, Honkaniemi H, Heshmati AF, . Unintended health consequences of Swedish parental leave policy (ParLeHealth): protocol for a quasi-experimental study. BMJ Open. 2021;11(6):e049682. doi:10.1136/bmjopen-2021-049682 34108172 PMC8191630

[19] Cnattingius S, Ericson A, Gunnarskog J, Källén B. A quality study of a medical birth registry. Scand J Soc Med. 1990;18(2):143-148. doi:10.1177/140349489001800209 2367825

[20] Massoudi P, Wickberg B, Hwang P. Screening for postnatal depression in Swedish child health care. Acta Paediatr. 2007;96(6):897-901. doi:10.1111/j.1651-2227.2007.00292.x 17537020

[21] Massoudi P, Hwang CP, Wickberg B. How well does the Edinburgh Postnatal Depression Scale identify depression and anxiety in fathers? a validation study in a population based Swedish sample. J Affect Disord. 2013;149(1-3):67-74. doi:10.1016/j.jad.2013.01.005 23499163

[22] Vägledning för barnhälsovården. Guideline for child healthcare. National Board of Health and Welfare. 2014. Accessed April 5, 2024. https://www.socialstyrelsen.se/globalassets/sharepoint-dokument/artikelkatalog/vagledning/2014-4-5.pdf

[23] Rikshandboken Barnhälsovård [The National Handbook for Child Healthcare]. Enskilda föräldrasamtal. Individual parental talks. 2019. Accessed August 12, 2024. https://www.rikshandboken-bhv.se/metoder--riktlinjer/enskilda-foraldrasamtal

[24] Massoudi P, Hwang CP, Wickberg B. Fathers’ depressive symptoms in the postnatal period: prevalence and correlates in a population-based Swedish study. Scand J Public Health. 2016;44(7):688-694. doi:10.1177/1403494816661652 27562827

[25] Asper MM, Hallén N, Lindberg L, Månsdotter A, Carlberg M, Wells MB. Screening fathers for postpartum depression can be cost-effective: an example from Sweden. J Affect Disord. 2018;241:154-163. doi:10.1016/j.jad.2018.07.044 30121448

[26] Ludvigsson JF, Andersson E, Ekbom A, . External review and validation of the Swedish National Inpatient Register. BMC Public Health. 2011;11:450. doi:10.1186/1471-2458-11-450 21658213 PMC3142234

[27] Wallerstedt SM, Wettermark B, Hoffmann M. The first decade with the Swedish prescribed drug register–a systematic review of the output in the scientific literature. Basic Clin Pharmacol Toxicol. 2016;119(5):464-469. doi:10.1111/bcpt.12613 27112967

[28] Ludvigsson JF, Svedberg P, Olén O, Bruze G, Neovius M. The longitudinal integrated database for health insurance and labour market studies (LISA) and its use in medical research. Eur J Epidemiol. 2019;34(4):423-437. doi:10.1007/s10654-019-00511-8 30929112 PMC6451717

[29] Dubbeldagar—vissa pappors väg in i föräldrapenningen? Double days—some fathers’ way into parental leave?. Swedish Social Insurance Inspectorate. ISF Reviews and Analyses. 2018. Accessed April 5, 2024. https://docplayer.se/105034515-Dubbeldagar-vissa-pappors-vag-in-i-foraldrapenningen.html

[30] Austin PC. An introduction to propensity score methods for reducing the effects of confounding in observational studies. Multivariate Behav Res. 2011;46(3):399-424. doi:10.1080/00273171.2011.568786 21818162 PMC3144483

[31] Ma L, Andersson G, Duvander AZ, Evertsson M. Fathers’ uptake of parental leave: forerunners and laggards in Sweden, 1993–2010. J Soc Policy. 2020;49(2):361-381. doi:10.1017/S0047279419000230

[32] Zacher Kjeldsen MM, Bricca A, Liu X, Frokjaer VG, Madsen KB, Munk-Olsen T. Family history of psychiatric disorders as a risk factor for maternal postpartum depression: a systematic review and meta-analysis. JAMA Psychiatry. 2022;79(10):1004-1013. doi:10.1001/jamapsychiatry.2022.2400 35976654 PMC9386615

[33] Lappegård T, Duvander AZ, Neyer G, Viklund I, Andersen SN, Garðarsdóttir Ó. Fathers’ use of parental leave and union dissolution. Eur J Popul. 2019;36(1):1-25. doi:10.1007/s10680-019-09518-z 32116476 PMC7018894

[34] Evertsson M, Boye K, Erman J. Fathers on call? a study on the sharing of care work between parents in Sweden. Demogr Res. 2018;39(2):33-60. doi:10.4054/DemRes.2018.39.2

[35] Blum S, Dobrotić I, Kaufman G, Koslowski A, Moss P. *19th International Review of Leave Policies and Related Research*. International Network on Leave Policies & Research; 2023. Accessed April 5, 2024. https://www.leavenetwork.org/annual-review-reports/review-2023/

[36] Flera förbättringar i föräldraförsäkringen. Several improvements in parental insurance. Government Offices of Sweden. 2023. Accessed April 5, 2024. https://www.regeringen.se/pressmeddelanden/2023/12/flera-forbattringar-i-foraldraforsakringen/

[37] Angrist JD, Pischke JS. Mastering ‘Metrics: The Path From Cause to Effect. Princeton University Press; 2014.

