# Supplementary material for: Postpartum Mental Health Care Use Among Parents During Simultaneous Parental Leave
Source: JAMA Netw Open. 2024 Oct 14;7(10):e2438755. doi: 10.1001/jamanetworkopen.2024.38755 (PMC11474414; doi:10.1001/jamanetworkopen.2024.38755)
Supplement: Supplement 1. — Figure. Flow Diagram of Study Population Selection Process eTable 1. Robustness Analysis: Propensity Score Matching Results of Association Between Simultaneous Parental Leave and Parents’ Mental Healthcare Use eTable 2. Robustness Analysis: Propensity Score Matching Postestimation Covariate Balance Summary eTable 3. Sensitivity Analysis: Simultaneous Parental Leave and Parents’ Mental Healthcare Use, Excluding Individuals With Mental Healthcare Use Before Birth eTable 4. Sensitivity Analysis: Simultaneous Parental Leave and Parents’ Mental Healthcare Use, Excluding Single and Non-Cohabiting Individuals in the Year After Birth [file jamanetwopen-e2438755-s001.pdf]

Supplemental Online Content

Honkaniemi H, Juárez SP. Postpartum mental health care use among parents during simultaneous parental leave. *JAMA Netw Open*. 2024;7(10):e2438755. doi:10.1001/jamanetworkopen.2024.38755

eFigure. Flow Diagram of Study Population Selection Process

eTable 1. Robustness Analysis: Propensity Score Matching Results of Association Between Simultaneous Parental Leave and Parents' Mental Healthcare Use

eTable 2. Robustness Analysis: Propensity Score Matching Postestimation Covariate Balance Summary

eTable 3. Sensitivity Analysis: Simultaneous Parental Leave and Parents' Mental Healthcare Use, Excluding Individuals With Mental Healthcare Use Before Birth

eTable 4. Sensitivity Analysis: Simultaneous Parental Leave and Parents' Mental Healthcare Use, Excluding Single and Non-Cohabiting Individuals in the Year After Birth

This supplemental material has been provided by the authors to give readers additional information about their work.

© 2024 Honkaniemi H et al. *JAMA Network Open*

eFigure. Flow diagram of study population selection process

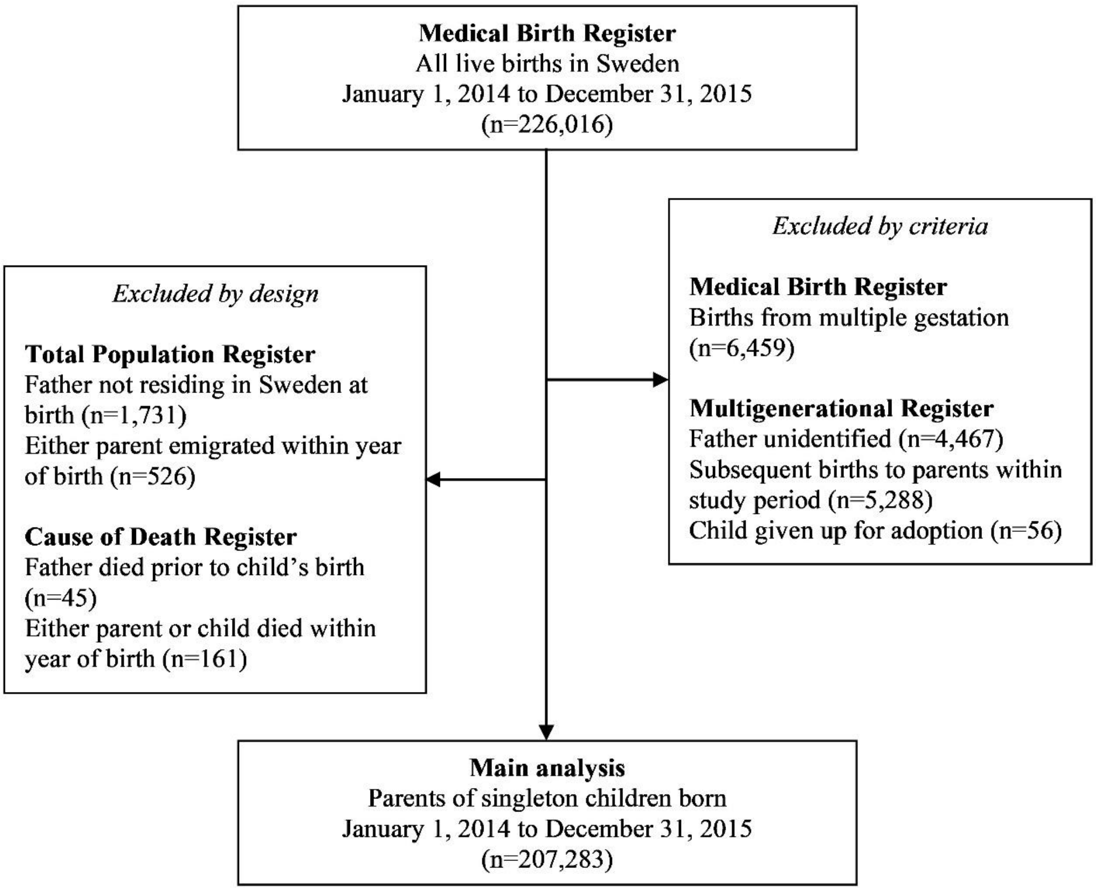

© 2024 Honkaniemi H et al. *JAMA Network Open*

eTable 1: Robustness analysis: Propensity score matching results of association between simultaneous parental leave and parents' mental healthcare use

	**1A. Mothers** (*n* = 207,283)	**1B. Fathers** (*n* = 207,283)
	**ATET Coeff. (95% CI)**	**ATET Coeff. (95% CI)**
**By simultaneous parental leave use** (ref. no leave use)		
**Outpatient mental healthcare visits**		
Substance use disorders	0.00019 (-0.00090-0.00127)	0.00101 (-0.00032-0.00234)
Mood and affective disorders	0.00091 (-0.00109-0.00291)	0.00090 (-0.00046-0.00226)
Stress-related disorders	0.00203 (-0.00042-0.00448)	0.00085 (-0.00096-0.00267)
**Psychotropic prescriptions**		
Antidepressants	**0.00468 (0.00190-0.00744)**	0.00192 (-0.00028-0.00411)
Anxiolytics	0.00125 (-0.00104-0.00353)	0.00233 (-0.00167-0.00233)

Abbreviations: ATET: Average Treatment Effect on the Treated; CI: Confidence Interval. Propensity score of treatment (simultaneous parental leave use) calculated based on both parents' age, educational attainment, labor income, nativity and individual pre-birth mental healthcare use. Individuals in treatment and control groups matched 1:1 based on propensity scores.

© 2024 Honkaniemi H et al. *JAMA Network Open*

eTable 2: Robustness analysis: Propensity score matching postestimation covariate balance summary

	Standardized Differences		Variance Ratio	
**Covariates**	**Raw**	**Matched**	**Raw**	**Matched**
Maternal age at childbirth				
20-24 years	0.1046026	0.004485	1.257853	1.008912
25-29 years	0.1075582	-0.0043810	1.082402	0.9973256
30-34 years	-0.0451626	-0.0009121	0.9685032	0.9993079
35-39 years	-0.1188984	-0.0002129	0.8040825	0.9995614
40+ years	-0.0926499	0.0046645	0.6262727	1.027434
Paternal age at childbirth				
20-24 years	0.0433755	0.0032819	1.197750	1.013042
25-29 years	0.1515441	0.0005119	1.227109	1.000583
30-34 years	0.0480978	0.0004684	1.034791	1.000308
35-39 years	-0.0757306	-0.0017811	0.9113156	0.9976340
40+ years	-0.1686745	-0.0009513	0.7232264	0.9978518
Maternal labor income				
Quintile 2	0.1257229	0.0022132	1.148164	1.002088
Quintile 3	0.1041686	0.0003716	1.122851	1.000364
Quintile 4	-0.0335183	0.0000873	0.9606733	1.000109
Quintile 5	-0.0916600	0.0082208	0.3985347	1.111878
Paternal labor income				
Quintile 2	0.1834180	0.0005626	1.215028	1.000471
Quintile 3	0.1783101	0.0012079	1.209410	1.001024
Quintile 4	-0.0954432	0.0000449	0.8887657	1.000062
Quintile 5	-0.0962191	0.0072487	0.3163931	1.124498
Maternal educational attainment				
Medium	0.1711813	-0.0046419	1.050775	0.9994806
High	-0.0814721	-0.0029859	0.9877121	0.9993103
Missing	-0.0891498	0.0148449	0.4822502	1.163913
Paternal educational attainment				
Medium	0. 185934	-0.0022879	0.9787685	1.000699
High	-0.1160584	0.0002795	0.9254148	1.000224
Missing	-0.0673884	0.0127306	0.5343541	1.154549
Maternal migrant status	-0.2461661	0.0045863	0.7282122	1.007679
Paternal migrant status	-0.2708600	0.0048378	0.7105476	1.008142
Maternal pre-birth mental health	0.0533824	0.0124166	1.129491	1.027528
Paternal pre-birth mental health	-0.0022871	0.0120970	0.9931726	1.037563

© 2024 Honkaniemi H et al. *JAMA Network Open*

eTable 3: Sensitivity analysis: Simultaneous parental leave and parents' mental healthcare use, excluding individuals with mental healthcare use before birth

	**3A. Mothers** (*n* = 182,388)	**3B. Fathers** (*n* = 189,851)
	**OR (95% CI)**	**OR (95% CI)**
**By simultaneous parental leave use** (ref. no leave use)		
**Outpatient mental healthcare visits**	**1.05 (1.01-1.10)**	**1.10 (1.05-1.16)**
Substance use disorders	1.06 (0.93-1.20)	**1.19 (1.07-1.32)**
Mood and affective disorders	1.02 (0.93-1.10)	**1.14 (1.02-1.28)**
Stress-related disorders	**1.11 (1.04-1.18)**	1.08 (0.99-1.17)
**Psychotropic prescriptions**	**1.08 (1.03-1.14)**	1.04 (0.98-1.10)
Antidepressants	**1.10 (1.04-1.17)**	1.05 (0.98-1.14)
Anxiolytics	**1.10 (1.02-1.18)**	1.02 (0.94-1.11)

Abbreviations: CI: Confidence Interval; OR: Odds Ratio. Estimates are adjusted as follows: By maternal and paternal age at childbirth; and maternal and paternal educational attainment, income, and nativity.

© 2024 Honkaniemi H et al. *JAMA Network Open*

eTable 4: Sensitivity analysis: Simultaneous parental leave and parents' mental healthcare use, excluding single and non-cohabiting individuals in the year after birth

	**4A. Mothers** (*n* = 184,475)	**4B. Fathers** (*n* = 184,475)
	**OR (95% CI)**	**OR (95% CI)**
**By simultaneous parental leave use** (ref. no leave use)		
**Outpatient mental healthcare visits**	**1.05 (1.01-1.09)**	**1.12 (1.06-1.17)**
Substance use disorders	**1.13 (1.00-1.27)**	**1.24 (1.12-1.37)**
Mood and affective disorders	1.03 (0.97-1.10)	**1.11 (1.01-1.21)**
Stress-related disorders	1.05 (0.99-1.10)	1.06 (0.99-1.13)
**Psychotropic prescriptions**	**1.04 (1.00-1.09)**	1.05 (0.99-1.11)
Antidepressants	**1.05 (1.01-1.10)**	1.04 (0.99-1.10)
Anxiolytics	1.01 (0.96-1.07)	1.02 (0.96-1.08)

Abbreviations: CI: Confidence Interval; OR: Odds Ratio. Estimates are adjusted as follows: By maternal and paternal age at childbirth; maternal and paternal educational attainment, income, nativity; and maternal or paternal mental healthcare use before birth.

© 2024 Honkaniemi H et al. *JAMA Network Open*
