# Supplementary material for: Postpartum Mental Health Care Use Among Parents During Simultaneous Parental Leave
Source: JAMA Netw Open. 2024 Oct 14;7(10):e2438755. doi: 10.1001/jamanetworkopen.2024.38755 (PMC11474414; doi:10.1001/jamanetworkopen.2024.38755)
Supplement: Supplement 2. — Data Sharing Statement [file jamanetwopen-e2438755-s002.pdf]

Data Sharing Statement

Honkaniemi. Postpartum Mental Health Care Use Among Parents During Simultaneous Parental Leave. *JAMA Netw Open*. Published October 14, 2024.
doi:10.1001/jamanetworkopen.2024.38755

Data
Data available: No

Additional Information
Explanation for why data not available: The data that support the findings of this study are available from The Swedish Public Health Agency but restrictions apply due to the sensitive nature of individual health data. Data are however also available from the authors upon reasonable request after ethical approval from the Ethical Review Board and with permission of The Swedish Public Health Agency. Data must be analysed at Stockholm University.
